# A socio-legal analysis of the Belgian protective legislation towards victims of aggravated forms of migrant smuggling

**DOI:** 10.1007/s10611-022-10029-y

**Published:** 2022-04-07

**Authors:** Roxane de Massol de Rebetz, Maartje van der Woude

**Affiliations:** Van Vollenhoven Institute for Law, Governance & Society, Leiden University, Leiden, The Netherlands

**Keywords:** Migrant smuggling, Human trafficking, Transit migration, Legal protection

## Abstract

As many scholars have shown, and other than what is suggested by their legal definition, migrant smuggling and human trafficking are not always easily distinguishable in reality. Acknowledging this grey area between the two phenomena, the Belgian legislature has introduced an alternative approach (referred to as ‘third-way approach’) which would offer migrants who have experienced ‘aggravated forms’ of migrant smuggling the same protective status that is usually strictly reserved for victims of human trafficking. Interestingly enough, migrants don’t seem inclined to make use of this procedure. Through a series of expert interviews, this article shines light on the perspective of key actors within the Belgian criminal justice system and migration control apparatus with regard to this third-way approach and its functioning in practice. In so doing, the article not only reveals how the proper functioning of this third-way approach is hindered by a series of organizational and institutional factors, but it also shows how the different actors are struggling with the inherent tension between the objectives of protecting state security and the protection of the needs of vulnerable groups in precarious life situations.

## Introduction

The so-called migration ‘crisis’ starting in 2015 has made clear that the principle of free movement of people, which results from the construction of the Schengen Area within the European Union (EU) as a space without internal border controls, knows it limits. Many countries in the Schengen area have amped up their border checks by introducing a range of bordering techniques (Van der Woude, 2020). These different formal and informal bordering practices have an impact on intra-Schengen secondary migration movements. The bordering practices within the Schengen Area and their consequences, notably on what is referred to as transit migration, have so far received limited scholarly attention (Barbero, 2020). The increased monitoring and management of mobility de facto turns different zones within the Schengen Area into ‘transit zones’ where irregular migrants on their way to their desired destination countries are stranded in limbo or ‘stuck in motion’ for a varying period during their journey. Numerous examples of these intra-Schengen transit zones can be found in the North of France, in Belgium as signaled above and in border zones between France, Spain, Italy and Switzerland (Tazzioli, 2019; Schapendonk, 2018; Myria, 2020). The vulnerable conditions of stuck/stranded individuals on the move have been reported inter alia by scholarship focusing on transit migration which, in this article, is to be understood as ‘phase of experienced immobility in process of movement in a specific migratory direction’ (Schapendonk, 2012: 579). Scholars have linked this experienced immobility to an increase of vulnerability. Indeed, once stranded or forced to move, migrants are more likely to turn to migrant smuggling networks, to take more dangerous paths, to increase their debts to reach their destination and to end up in exploitative conditions (O’Connell Davidson, 2013; Kemp, 2017; Brigden & Mainwaring, 2016). Whereas the concept of transit migration and the notion of being stranded, as experienced by migrants, is mostly used in the context of describing how *external* borders are ‘blocking their [migrants, MW] onwards movements’ (Schapendonk, 2012: 580), this article uses the concept to illuminate the equally problematic reality of the blocking effects of *internal* border checks between Schengen states (e.g., Van der Woude, 2020) and governmental practices policing migrants (e.g., Tazzioli, 2019; Edmond-Pettitt, 2018).

Due to its geographical position, its sea connection with the United Kingdom (UK) and its dense road network to reach different international borders, Belgium is historically known to be attractive for irregular border crossings (Derluyn & Broekaert, 2005; Melis & Van Gelder, 2017). Moreover, the role of the capital Brussels as a ‘migrant smuggling hub’ has been highlighted in the scholarship (e.g., Leman & Janssens, 2015). The increase of migratory pressure in the EU since 2015 has led to a rise of secondary migration movements and gatherings of new migratory groups in the Belgian territory, particularly since the dismantling of the Jungle of Calais by the French authorities in 2016 (see Myria, 2020). The neighborhood of the Brussels North Station and the Maximilian Park have become gathering places for many individuals in transit who are awaiting an opportunity to reach the UK (Myria, 2020). Consequently, migrant smuggling and issues surrounding transit migration received substantial media and governmental attention (e.g., Framework Note on Integrated Security 2016-2019). Already in 2015, a ‘*transit migration’* taskforce was created, and the phenomenon became a federal priority in 2018 with the establishment of a transit migration plan (Belgian House of Representatives, 2018). These actions can be seen as response to growing political concerns on the emergence of new camps in Belgium and the role of the capital of Europe as a ‘pit stop’ or ‘mini-Calais’ used by migrants to reach the UK (Vandevoordt, 2021). The (controversial) neologism ‘transmigrant/transitmigrant’ does not refer to a formal legal category and was created to make a distinction between the migrants who wish to establish themselves permanently in Belgium and those who are only passing through the territory to reach the UK (Flemish Dictionary).

As many scholars have shown, other than what is suggested by their legal definitions, in reality, migrant smuggling and human trafficking are not always easily distinguishable (e.g., Dandurand & Jahn, 2020; Brunovskis & Surtees, 2019; Baird, 2016; Van Liempt, 2011; O’Connell Davidson, 2013). While complying with the most important European Union and Council of Europe normative frameworks adopted to deal with human trafficking and migrant smuggling and the established dichotomy between the two crimes since the adoption of the United Nation Convention against Organized Crime (UNTOC) and its two additional protocols (Palermo Protocols) in 2000,^1^ the Belgian legislation has gone further when it comes to the protection of victims of human smuggling by introducing the *‘third-way approach’.*^2^ When ‘aggravated forms’ of migrant smuggling can be proven, victims of migrant smuggling have access to the protective legal status that is usually strictly reserved for human trafficking victims. However, based on the numbers provided by the Belgian National Rapporteur for human trafficking and migrant smuggling (hereinafter Myria, 2018, 2019), very few victims of aggravated forms of migrant smuggling seem to end up with this protective status. In 2017 and 2018, only 19 victims were registered in the procedure while respectively 476 and 535 migrant smuggling cases entered the public prosecution office in 2017 and 2018 (Myria, 2018, 2019).

This observation shows how the law in books – the third-way approach - in practice does not seem to have the intended effect which, following the tradition of ‘gap studies’ (Gould & Barclay, 2012) calls for a closer analysis. As Schrooten (2018) noted, with (trans)migration issues becoming more complex and covering many more places than a straightforward path from a country-of-origin A to a destination country B, it is important to get a better grasp of this gap in victim protection. By drawing on a combination of (legal) document analysis and semi-structured expert interviews with respondents responsible for the implementation and enforcement of the third-way approach (see section 3), this article aims to further illustrate and explain this gap. Besides, the article also offers a critical examination of the growing presence of human rights and humanitarian ideals in border policing by addressing the challenges of policing humanitarian borderlands. The term ‘humanitarian borderlands’ refers to highly conflicting environments, where the objectives of protecting state security clash with the needs of vulnerable groups in precarious life situations (also see Aas & Gundhus, 2015).

## Policing humanitarian borderlands

Scholars working on migration and border control have increasingly been able to shed light on the complex workings of the migration control apparatus and the actors within it. In particular, the decisions of frontline agents as quintessential street-level bureaucrats have received scholarly attention demonstrating how immigration bureaucrats create de facto policies through their discretionary decision-making (Calavita, 1992; Heyman, 2009). Yet, as noted by Vega (2018), although this more traditional gap study approach that focuses strongly on the question *how* state agents wield their discretionary power has led to important insights on, for instance, the growing merger of crime control and migration control, it risks obscuring the ‘moral economy’ of these bureaucrats’ work lives. Although state agents indeed play a lead role in ‘translating’ immigration laws and policies into practice, it is crucial to keep sight on the fact that their ‘performances occur largely behind the closed doors of guarded government bureaucracies’ (Vega, 2018: 2546) and the fact that their decisions are shaped by the ‘moral economy’ (Fassin, 2005) of their work lives. In other words, in understanding actions and perceptions of (frontline) state agents, one needs to take into consideration the economy of the normative values and ideals of this particular group. This call to look beyond the individual of the street-level agent to better understand the complex variety of factors and actors that influence their decision-making process resonates with the call for what Van der Woude (2016), following Hawkins (1995), has named a more ‘holistic approach’ towards understanding (discretionary) decision-making practices by street-level state agents. Focusing on the French case, Fassin (2005) uses the notion of moral economy to unpack the tension between what he calls ‘discourses and practices of compassion and repression’ in the policies of immigration control.

This tension is also acknowledged by Aas and Gundhus (2015) who observe two distinct developments in European security policy and practice: on the one hand they note an intensification of migration and border control as well as a securitization of migration in Europe, but at the same time, they also see how a growing prominence is given to human rights and humanitarianism within international and domestic governance (van Zyl Smit & Snacken, 2009). While researching how this tension influences the way Frontex officials perceive and implement their duties, the authors observe how – through the actions and perceptions of state agents – migrants are objects of a ‘minimalist biopolitics’ (Walters, 2011) which they understand as a form of political reason that ‘employs the language of humanity and humanitarian assistance and usually characterizes the activities of national and international humanitarian actors as well as aspects of policing and migration control’ (Aas & Gundhus, 2015: 12). Although not seen as ‘proper’ security subjects of the state, and thus deserving of the state’s full protection, migrants are nevertheless seen as deserving compassion and humanitarianism. This language of compassion is not free of, nor outside of, politics according to Fassin (2011). He argues that the politics of compassion or humanitarianism is a salient mode of governance that concerns populations in situations of precariousness. And, when there are politics at play, there is at least reason for some healthy skepticism as to what is driving this shift towards a more humanitarian approach and who is to gain from such a shift. Bosworth (2017) speaks of the development of ‘penal humanitarianism’ with which she refers to the way in which human rights talk and humanitarianism can also be used to justify and legitimize an increase in the state’s penal power at the same time. While being careful of dismissing humanitarian sensibilities and logics as fraudulent rhetoric for a will to power, it is important to critically reflect upon them in the light of the Janus-face quality that has been attributed to various Western-European self-proclaimed ‘human rights champions’ (see Franko et al., 2019). The third-way approach is meant to offer a stronger level of protection to victims of migrant smuggling and can therewith be seen as a more humanitarian approach as well. This makes it interesting to see how key actors working in the criminal justice system and the migration control apparatus (broadly understood) perceive the approach and its practical application.

## Methodology

This research follows a single case study research design, with the Belgian third-way approach as its core. This article is primarily based on semi-structured expert’s interviews conducted between 2018 and 2019, supplemented by various bureaucratic (legal) documents, academic literature, and investigative journalist pieces. The data was supplemented by scholarly articles, NGO reports and newspaper articles to obtain the most multi-faceted results. In line with the multidisciplinary and integrated Belgian approach (see Group of Experts on Action against Trafficking in Human Beings within the Council of Europe– hereinafter GRETA 2013), the sample of interviewees reflects the fragmented Belgian institutional landscape as much as possible. In total, 15 interviews were conducted with the key members of distinct organizations which includes the public prosecutor office from different judiciary districts, the federal Prosecutor office, the federal police, the local police, the specialized reception center for victims of human trafficking and victims of aggravated forms of migrant smuggling (hereinafter: NGO), the Ministry of Justice, and the Foreigner’s Office. To have a broad overview of the phenomenon and to include more diverse (and critical) lenses, an in-depth interview was also conducted with an investigative journalist who produced an extensive investigation on the topic in 2018. Another interview was conducted in 2021 with a project manager of the Citizen Platform ‘BXLRefugee’ which, since 2016, has provided direct humanitarian assistance to (among other individuals) migrants in transit in Belgium.

To have a better understanding of the social reality and the shaping of social practices, qualitative interviews with experts are considered essential to gather experiences and insights on the way the migrant smuggling phenomenon is dealt with and perceived in Belgium (Edwards & Holland, 2013; Döringer, 2021). This article, which follows in that tradition, is part of a broader (comparative) research project on the intersection between migrant smuggling and human trafficking in the Schengen area. Respondents were for instance asked about their view on the links between migrant smuggling and human trafficking, the evolution of both phenomena, the functioning of the Belgian approach as well as their specific role in the procedure, their training and experience, their relationship with other actors from distinct organizations, the challenges they encountered when performing their tasks. Yet, the scope of this article is more specific as, the lack of protection of aggravated victims of migrant smuggling emerged quickly from the data. Nevertheless, the information gathered more broadly sheds light on the reasons why the third-way approach was scarcely used in practice.

Although interviews are a powerful tool to expand the scope of understanding of a particular phenomenon, they also come with important limitations. Perceptions are an *understanding* of the reality, and they can be subjective and susceptible to change over time (Alshenqeeti, 2014). Interviews generate perceptions, which do not necessarily reflect the ways in which respondents make decisions in reality. While expert interviews are subject to methodological debates, they are considered useful due to the expert’s professional, technical, institutional/organizational, and interpretive knowledge acquired during the course of their career (Bogner et al., 2018). These distinct criteria also refine what is to be understood by ‘expert’s knowledge’ and they were subsequently used to select the expert respondents to be interviewed.

Specialized prosecutors who are in charge of directing and monitoring investigations for human trafficking and migrant smuggling cases were selected. They follow a continuous training on both phenomena and act as liaison with other relevant actors competent to deal with trafficking and smuggling. We also interviewed respondents from the Belgian police, which is an integrated police service since 1998, structured at two levels: the local level and the federal level (De Valkeneer, 2016). Both levels work on the basis of complementarity, however, they have different missions. The federal police has specialized tasks, which include migrant smuggling, and acts on the basis of the principle of subsidiarity and specialty. The federal police has tasks of both administrative and judiciary nature in specialized areas, and is competent with regard to phenomena that exceed the local level. The main missions of the local police encompass core policing missions having both an administrative and judiciary nature (e.g., local investigation, public order, road safety). The local police is considered to be the eyes and ears on the ground, responsible for the first line detections (Demeester, 2015 cited in Myria, 2015). As de Valkeneer (2016) mentioned, there is in fact no strict partition when it comes to these missions. Hence, regarding migrant smuggling, both the local and the federal police can be competent, even if the latter falls a priori under the specialized competence of the federal police. Lastly, reception centers for victims (NGOs) play a central role in the managing of migrant smuggling and migrant trafficking, as they interact on a regular basis with police officers and specialized prosecutors (see GRETA, 2013). For instance, when a police officer suspects an individual to be a victim of trafficking and/or aggravated form of migrant smuggling, the first call to make is to the reception center and not the prosecutor. We therefore also spoke with a director of one of the three main reception centers for victims of human smuggling and human trafficking in Belgium.

The interviews lasted between 105 and 180 min and were audio recorded, transcribed, and subsequently coded with a coding software *Atlas.ti*. Interviews were carried out in French, Dutch and English or a combination thereof. The quotes singled out in the article were translated to English by the authors (one is a Dutch native speaker) when they were not originally expressed in that language.

## Dealing with migrant smuggling in Belgium

### Migrant smuggling and human trafficking as distinct crimes

Following the implementation of international and European instruments, as is the case in several other European countries, human trafficking and migrant smuggling are considered distinct offences in Belgian law since 2005. The offence of human trafficking is enshrined in the criminal code (art. 433*quinquies*) and the migrant smuggling offence can be found in the Law of 15 December 1980 concerning the access to the territory, stay, settlement and removal of foreigners (art. 77*bis* et seq.). Migrant smuggling can be defined as ‘*the act of facilitating, in some way or another, be it directly or by an intermediary, the unauthorized entry, transit, or stay of a non-EU citizen into or through a EU member state, in violation of state law, directly or indirectly, for financial gain*’. The legal dichotomy is based on the presence/absence of exploitation, the necessary crossing of a border for migrant smuggling, the legal interest protected by the offences, namely the protection of the integrity of the national borders for migrant smuggling and the protection of human dignity for human trafficking, and indirectly the presence of consent in migrant smuggling cases. Nonetheless, Belgian scholars (e.g., Derluyn & Broekaert, 2005), practitioners (e.g., Algoet, 2018) as well as policy makers (National Action Plan against Human Smuggling, 2015-2019) report on the complex intertwinement of and ‘cause and effect’ relationship between the phenomena in practice. The Correctional Tribunal of Louvain (12 May 2015) considered the boundary between the phenomena ‘*so thin that one can turn into the other once the freedom of action of the victim* [of migrant smuggling, MW] *is put in peril*’.

### Acknowledging and harmonizing aggravated circumstances

The Law of 10 August 2005 reforming the legal frameworks on both human trafficking and migrant smuggling harmonized the aggravating circumstances for both crimes. According to the legislature, consistency had to be maintained regarding the dramatic consequences of these crimes (Huberts, 2006). The aggravating circumstances can allow for the victim the possibility to receive a protective status, which is usually exclusively reserved for victims of human trafficking. The protective status and the procedure for its allocation is thoroughly described in the Circular as putting in place the multi-disciplinary cooperation regarding the victims of human trafficking and aggravated forms of migrant smuggling (COL 5/2017). It is important to highlight that each actor (e.g., reception centers for victims, prosecutors, local and federal police, labor inspectorate, Foreigners’ Office) has a specific role in the procedure. The reference magistrate in human trafficking and/or migrant smuggling (see section 3) has a monopoly on the formal decision to give the provisory status to the victim, which also explains the importance of gathering their perspective on these phenomena. Following Directive 2004/81/EC, a reflection period of 45 days and a first temporary then permanent residence permit under several conditions can be granted to the victim. Regarding the conditions, the provisory status can only be granted if investigation/judicial proceedings are ongoing (determined by the prosecutor). The (presumed) victims are required to collaborate with the authorities by making relevant declarations, cut ties with the presumed perpetrators, and be accompanied by one of the three reception centres for victims (for the full list see COL 5/2017 or GRETA, 2013). If the victim’s declaration leads to a conviction of the perpetrator or if the prosecutor chooses to push charges based on aggravated forms of migrant smuggling, the victim can obtain a permanent residence permit (GRETA, 2013, 2017).

The complete list of aggravating circumstances can be found in the article 77*quater* of the Law of 15 December 1980. Of particular relevance for this article are the following: the smuggling offence being committed by abusing the vulnerability of an individual resulting from its irregular or precarious administrative situation, its precarious social situation, state of pregnancy, illness or physical/mental impairment in a way that the individual has no real and acceptable alternative but to submit him/herself to the abuse; the direct or indirect use of fraud, violence, threats or any form of coercion as well as abduction, deception and abuse of power; the endangerment of the victim’s life (deliberately or through gross negligence) and an incurable illness/work disability resulting from the offence. While many forms of violence or threats do not call for clarifications, it is important to note that debt bondage is considered to be a form of coercion (Clesse, 2013). Moreover, placing individuals in refrigerated trucks or overheated ones without water is considered by the jurisprudence as gross negligence which endangers the lives of these individuals (see the case law highlighted in Clesse (2013)). Based on these clarifications, it can be concluded that the situation of migrants in transit found in lorries or in parking areas are most likely to fall within the ‘aggravated’ migrant smuggling category. Nevertheless, as mentioned in the introduction, few of these victims use the protection mechanism in place (Myria, 2018, 2019). While Belgium is often mentioned in scholarship for its well-integrated multi-disciplinary approach against human trafficking (GRETA 2013, 2017), the Belgian approach to deal with aggravated forms of smuggling is relatively unknown (Boels & Ponsaers, 2011; Clesse, 2013).

## The third way approach through the eyes of state agents

### The legal reality of the third-way approach

As the third-way approach was introduced partly also to acknowledge the interwovenness of human trafficking and human smuggling, and thus to acknowledge that both phenomena in practice might not be as easily distinguishable as suggested by the seemingly clear-cut legal categories, we first asked respondents how they perceived this. Interestingly, the majority of the respondents considered the legal dichotomy between human trafficking and migrant smuggling to be evident. In their answer, most respondents referred in a very legalistic manner to the distinctive elements highlighted in 4.1, such as the presence of consent and the absence of the exploitation dimension in migrant smuggling cases. Prosecutor 2 was concerned about the implications of letting go of this legal distinction, even if this distinction does not reflect the messiness of reality: ‘*If you start mixing them, you’re facing the risk to have acquittals, as you have two totally distinct criminal offences.’* They continue by explaining that this would also concern cases of migrants being exploited in the Maximilian Parc because ‘*the people who pick up undocumented individuals there and exploit them, for them it’s solely human trafficking, it’s not smuggling. And then I cannot prosecute my smuggler* [referring to the vignette presented to the respondent where the smuggler is the defendant, RM]*, for human trafficking because he has no idea of what’s going on site* [in the Maximilian Parc, RM]*’*.

Nonetheless, while acknowledging and supporting the legal categorization of migrant smuggling and human trafficking the majority of respondents also recognized the potential continuum as well as the intrinsic link existing between the two phenomena, notably when it came to debt-bondage and vulnerability (see O’Connell Davidson, 2013). As two attachés of the Ministry of Justice state:

*‘For us the legal dichotomy is clear, but among the smuggled individuals, you can find elements of human trafficking that happened. (…) And there are many smuggled individuals who take on debts to come to Europe. In fact, the situation of smuggling creates a situation of vulnerability. There is already a situation of vulnerability from the start, but it is accentuated by the smuggling or by the fact that they paid dearly their journey and then have to reimburse the debt. (…) So, you have victims that once they arrived, because of the fact that they were placed in a situation of vulnerability or due to their irregular situation, can be more easily recruited by people who will exploit that vulnerability*.’

Regarding the secondary migration movements within the Schengen Area, Prosecutor 1 called specific attention to the notion of vulnerability by explaining that authorities ‘*will have to be attentive to human trafficking, because, in my opinion, there are going to be a lot of people wandering in Europe because we’re seeing these secondary movements (…) and these people could be victims of human trafficking as they don’t have anything and they are extremely vulnerable’*. Prosecutor 1 also raised a pragmatic issue when tackling a case linking both phenomena in which individuals were stuck and exploited in Belgium not knowing if they had to ‘wait’ for weeks/days/months to reach their desired destination country. The difficulty of finding the evidence to prosecute on both accounts was signaled. The magistrate identified these situations as challenges that prosecutors will have to face in the coming years with the millions of people who moved towards Europe since 2015 and were severely exploited along the way. Due to rules of jurisdiction, even if they can be considered as human trafficking victims, when they are in Belgium, authorities can only focus on the migrant smuggling dimension as the migrants were not exploited in the Belgian territory and/or by Belgian nationals. Therefore, instead of being seen as potential victims requiring enhanced protection, they will most likely be treated as migrants in irregular stay in the territory that need to be returned.

Regarding evidence issues, the Director of one of three reception centers for victims also referred to the situation of ‘*transmigrants*’ exploited ‘*temporarily*’ by being picked up in the Maximilian Park. While the Director said that they ‘could be’ considered as victims of human trafficking, (s)he also stated that it is difficult to really call it trafficking due to the short-lasting duration of the exploitation, which is at odds with the human trafficking business model based on the long-lasting exploitation of an individual. Pragmatically, and reflecting on the possibility of securing a conviction for human trafficking with the evidence requirements, the Director explained that for cases of short-term exploitation *‘you’ll never get that convicted (…) because you need to prove that it happened for starter’*. Yet, when looking at article 433*quinquies* of the Criminal Code criminalizing human trafficking, no mention of temporality can be found. Pragmatic arguments were also raised by several respondents regarding the challenging gathering of evidence. The NGO Director explained bluntly that even if you have individuals who are completely abusing the vulnerable situation of those who want to reach the UK, the migrant exploited in a temporary manner will never admit the fact that he was trafficked, as he got 20 euros for a day of work instead of nothing.

In answering the questions about the potential links between two phenomena, several respondents spontaneously mentioned the existence of the protection for victims of aggravated forms of migrant smuggling. They see the protective framework as a mitigator of the strict legal dichotomy established between the phenomena. When asked to reflect on the term ‘aggravating circumstances’ and if he could find them in the specific context of migrants in transit, a Federal Police lead Investigator in the migrant smuggling field responded: ‘*the aggravated circumstances, I have them immediately’*. Basing his argument on the law and the jurisprudence developed on the abuse of a situation of vulnerability, he stated, ‘*the fact that he is staying in the country illegally is an aggravating circumstance as far as I’m concerned*’. Regarding evidence of the abuse, the latter is ‘*easily proven (…). If someone is smuggled, he is abused because he pays way too much for what he gets*.’ Confirming the development of the scholarship and the jurisprudence highlighted in section 2.2, Prosecutor 4 went even further:

*‘If you illegally transport a person to England, it is automatically someone who is in a vulnerable situation. I don’t know a single person in an illegal situation who isn't in a vulnerable state. So automatically you have the aggravating circumstance of vulnerability.’*

This last quote illustrates how, in theory, the legal threshold to be considered as a potential victim of aggravated forms of migrant smuggling is not unreachable. Besides, it also shows that in practice, this third-way approach could indeed be seen and used as a mitigator for the strict dichotomy in place between migrant smuggling and human trafficking. Yet, as the following sections illustrate, when asked about the application of the third-way approach in practice, in particular the extent to which victims of smuggling are better protected under this approach, the respondents seem to be less able to paint a clear picture.

### The third-way approach in practice

When asked about victims’ protection all respondents pinpointed that ‘*the system is not working*’ (Prosecutor 2). By looking at the numbers, one of the respondents noticed that the ‘*in-between status for victims of migrant smuggling is never used in practice*’ (Federal Police Investigator 1). Different reasons were highlighted to explain this. All respondents mentioned that the victims of aggravated forms of migrant smuggling do not wish to settle in Belgium and want to reach the UK at all costs. As expressed by Prosecutor 1: ‘*When you already came 3000 km through the desert, it is not the last 500, 1000 or 30 km that will make you change your mind, even if we explain them that in Belgium, they will have rights and papers’.* The UK being seen as an Eldorado was often mentioned as a pull factor for migrants. The common points that are often identified as attractive are the following: the presence of large migrant communities which facilitate blending in, the language, the absence of identification requirements, the presence of an important shadow economy and the rumors that the British do not apply the Dublin III Regulation (Foreigner’s Office respondents 1 and 2, Prosecutor 1 and 4 - see also the report of Myria, 2020 comparing Belgian and British legislations). After all, ‘*the English themselves say: our country is the best country to disappear*’ (Prosecutor 1). It is worth mentioning that frustration was expressed about the lack of action undertaken by the UK to counter the erroneous information on the non-application of the Dublin regulation in their country to the irregular migrants staying or passing through Belgium: ‘*they say that’s your problem, we have our problems*’ (Prosecutor 4). Whereas these perceptions were gathered between 2018 and 2019, the recent and increasing perilous border crossing by boat of the English Channel leading to dramatic consequences for migrants and the reaction of French and British authorities pointing fingers at each other on who is to blame for these incidents highlight similar dynamics (The Guardian November 2021).

Furthermore, the Dublin III Regulation is identified as a major factor guiding the victim’s decision to not enter the protective procedure offered through the third way approach due to the ‘*fear to be sent back to Italy if they start the procedure in Belgium*’ (Foreigner’s Office Respondent 3). The same respondent mentioned that because the goal of irregular migrants is not to stay in Belgium, he believes that ‘*the threshold is too high to enter the procedure’*. With this, he refers to the conditions that need to be met to start the procedure and to receive the status. All of this is too demanding in light of the needs and desires of the migrants. Indeed, several magistrates confirmed that ‘*the help* [the protective status, RM] *is not materially possible*’ as the ‘*victims will have to install themselves in a reception center, they will have to collaborate* (with relevant declarations), *they will have to cut all the links with their smugglers*’ (Prosecutor 2). In other words, there is much to lose, whereas it might not be clear for migrants what there is to gain. Another Prosecutor (3) explained the ‘*reluctance*’ of victims to turn against their smuggler to enter the procedure as ‘*they only want one thing: to start again*’, and thus they don’t want to burn any bridges with their one connection to ‘Eldorado’. The lack of interest to incriminate one’s smuggler can also be explained by two other reasons: the fear that migrants have for either their own or their family’s lives and a change in the financial modus operandi of migrant smuggling in the last years. Prosecutor 1 explains that, in some cases, migrants make a deposit somewhere and it is only once they have arrived at their desired destination country that the money is liberated, which explains the lack of incentive to denounce one’s smuggler *en route*. The attachés from the Ministry of Justice also mentioned the trust existing between the smugglers and the migrants, which explains why police officers can ‘*face a brick wall’* when trying to convince the migrants to enter the procedure.

Regarding the collaboration with the authorities, any information, however small (e.g., the place where they met their smuggler), can be considered sufficient for police officers to fulfil the ‘relevant declarations’ requirement (Federal Police Investigator 1) that, as explained in section 4, is necessary to show that migrant is ready to break ties with the smuggler/smuggling organization and thus to trigger the third way approach. However, migrants are hard to convince to do so. According to the respondent, in comparison with victims of human trafficking, because they are staying in the territory, migrant smuggling victims are only passing through, so he ‘*only has one or two chances to persuade them*’. This persuasion is not easy as the person has to want it her/himself, and the respondents of the Foreigner’s Office (2 and 3) mention that even when you bring interpreters into the mix, ‘*they’re simply not open to it*’. As framed by the Director of the NGO, ‘*victims don’t want to enter the status because then they appear on the grid, and they do not want to be on the grid’*. In short, as explained by Prosecutor 4, ‘*we have nothing to offer to these people’*, which can be interpreted as him being conscious of the limitations of the protective status in practice. Prosecutor 4 explained that when they’re asking migrants ‘*to leave the territory*’, migrants sometimes reply in a humoristic manner: ‘*we would love to leave the territory, but preferably at the North Seaside*’.

Based on these insights, it is of interest to critically reflect on the fact that respondents, when explaining why the protective status is scarcely granted, are mostly pointing at migrants’ lack of interest in entering the procedure. One can question the extent to which this is a fair depiction of the reality. While several other reasons touching upon the complexity of the institutional framework, organizational culture and capacity issues will be further developed in section 6, the lack of information or the inadequacy of information received by migrants must also be underlined as potential factors to consider explaining the ineffectiveness of the third-way approach. That information issue was signaled by the investigative journalist and further confirmed by the project manager of the citizen organization ‘BXLRefugee’. In addition, the Permanent Oversight Committee of the Police Service also launched an official investigation on police control and detention of ‘transmigrants’ taking place during large-scale administrative arrest operations (often in parking areas along highways). The report showed that during these operations, the police forces took *little to no account* of the guidelines (of criminal policy) on human trafficking, migrant smuggling as well as the protective status reserved for victims of trafficking and aggravated forms of smuggling (Comité, 2019).

## A more complex answer: Institutional forces and dynamics at play

The ineffectiveness in practice of the third way approach cannot only be explained by the reasons highlighted above. Respondents drew attention to the fact that dealing with migrant smuggling and the protection of its victims intersects with other ‘fights’ the state is facing: the fight against irregular migration, against human trafficking and the maintenance of national security and public order involving a multitude of actors with distinct (political) agendas that can clash at times. Moreover, issues of police capacity and training/sensibilization, national and international institutional collaboration as well as victim’s information were mentioned by the respondents as important challenges which prevent finding structural solutions to deal with the complex phenomenon. These factors will be further unpacked in this section.

### Overlapping and clashing competences

The complexities caused by overlapping competences and related responsibilities of distinct actors can best be illustrated by taking a closer look at the situation of the migrants gathered in the Maximilian Park and the nearby Brussels North train station, waiting to go to highway rest areas in the Belgian territory to try their luck at reaching the UK. In a nutshell, on the federal level, the government is responsible for migration and asylum policy. The Foreigner’s Office is responsible for the entry and stay of foreigners whereas Fedasil is the federal agency responsible for the applications for international protection. Lastly, the federal police (judiciary branch, the railway police and the road police) is responsible for different tasks such as dismantling of smuggling networks, the safety and security of the station and the railway clients and road safety on the highways. At the local level, the Brussels North zone is under the authority of two local police districts of Brussels. Local police officers are responsible for maintaining security and public order on the local level, but they can also participate in migration checks coordinated by the federal police and the Foreigner’s Office. Lastly, various NGOs are active in the Maximilian Park to provide humanitarian assistance and care to irregular migrants (see, for instance, Plateforme Citoyenne/BXLRefugee). If migrants are moving along Belgian highways, other local police districts, judiciary federal police units and political actors such as the Governor of the Province and the mayors under his authority concerned with the issue can become competent and responsible to act.

The complexity of the Belgian institutional system and the (increased) fragmentation of the state action among a multitude of actors acting at distinct levels is not new and was already pinpointed as an important factor to consider when looking at the effectivity of public policy. Numerous and various examples on how institutional complexities, particularly in the case of Brussels, can hinder the effectiveness of public state action can easily be found in the scholarship and range from the issue of homelessness (Malherbe et al., 2019), the integration of newly arrived immigrants in the territory (Xhardez, 2016) to the general administration of primary care during the first wave of Covid 19 (Jamart et al., 2020).

The Federal Police Investigator and the respondents of the Foreigner’s Office regularly mentioned the different agendas of each actor clashing with one another. For example, as they explained, whereas the judiciary federal police might find it convenient to have migrants gathered at the same place, as it is convenient for their investigation and observations of potential smugglers, this might cause a problem for the railway police, who act with the railway customer’s safety in mind. Similarly, when the local police take on administrative police tasks (control and arrest of individuals in irregular situation), they are acting under the mayor’s authority who is concerned with citizens’ complaints about, amongst other things, the safety and cleanliness of the Maximillian Park and the surrounding area that they would like to have available for their children. For political ideological reasons, the different mayors competent for the area can refuse to enforce migration checks to arrest irregular migrants, which then clashes with the recommendation taken at the federal level to instead engage in actions of maintenance of hygiene and safety (Foreigner’s Office respondent 2). At the local level, the area of Brussels North is under the authority of two local police districts of Brussels (Brussels North Zone composed of 3 municipalities and the Brussels Capital/Ixelles Zone composed of 2 municipalities), which consequently depend on the authority of different mayors. The role of the mayors is essential (see 6.2) as the administrative police at the local level fall under their competences. As respondents explain, by prioritizing the cleanliness of the park over performing migration checks, Maximilian Park has obtained the status of being a ‘*rest area’* and thereby a ‘*pull factor’* for migrants. They come to the park to receive basic assistance and care by NGOs before undertaking the final leg of their journey. According to several respondents, this situation upsets the mayors and the Governor in the West-Flanders region where constant judiciary and administrative actions are undertaken to dismantle migrants’ smuggling networks and prevent the creation of ‘*mini-Calais’* in their territory (Federal Police Investigator; Investigative Journalist; Foreigner Office respondents 2 and 3, Prosecutor 4). The respondent of the Foreigner Office (2) expressed his frustration regarding the absence of ‘*unicity in the decision-making*’, as he explains:

‘*It can’t be that transit migrants in the territory of the capital of Brussels are left alone by the authorities, whereas the municipality next door pursues an active persecution policy of transmigrants. This is inefficient. It leads to an enormous, how shall I put it, misallocation of people and resources.*’

### Capacity and the need for sensibilization

The former quote highlights the issue of police capacity which plays a role at both the federal and local level. The majority of respondents signaled that in the past years, the federal police level was ‘*emptied of its substance to give the priority to terrorism*’ (Prosecutor 3). With this, the Prosecutor refers to the fact that, in response to the terrorist attacks of 2016, a large portion of federal police officers was moved to the terrorism unit.

While the respondents understand that terrorism is important, they are concerned about the fact that ‘*the capacity didn’t return (…) and that very experienced detectives just left and by now, are almost ready to retire but they were never replaced by detectives of the same value, which is crippling* [the organization, MW] *basically*’ (NGO 1). Regarding the local police districts, ‘*the mayors are the one deciding on the priorities and victims of migrant smuggling and human trafficking are not the priority in comparison to thefts and road safety*’ (Prosecutor 1). While migrant smuggling can be a political priority where structural synergy between the federal and the local police can be observed, it is not systematic and varies from one zone to another (Federal Police Investigator, Prosecutor 4). Besides, the Federal Police Investigator explained that arresting someone without papers can lead to two distinct procedures: an administrative one on the legal status of the person and a criminal procedure that ‘*starts as soon as you find aggravating circumstances*.’ The time-consuming nature of the procedures and the weak ‘*return on investment’* leading to the police’s frustration was highlighted by the majority of respondents.

‘*There are local police districts saying ‘we saw 30* [migrants, RDM] *on the parking, we looked elsewhere and when we turn back, they were gone. I can understand, when you’re only two* [frontline police officers, RDM] *and you have 30 migrants, you need to identify them, to take the fingerprint, to contact the Foreigner’s Office. With only two of them, it can take up to 24 hours’* (Prosecutor 1). To which a policy officer from a local police district (1) replied during the interview: ‘*all of that so that the Foreigner’s Office can deliver an order to leave the territory*’. As the capacity of administrative detention centers to place migrants is limited, migrants receive this administrative order in the majority of cases, which they throw in the nearby trash to then call their smugglers and start their journey again (Investigative Journalist; also see De Ridder & van der Woude, 2016).

This quote by Prosecutor 1 is crucial in understanding the clash of priorities, the lack of capacity and the frustration of state agents when dealing with migrant’s smuggling. For the prosecutors and the federal police, any piece of information is relevant for the investigation and ideally, all migrants’ phones that are found should be searched, but for the chief of the local police zones and the mayors, it is seen as a waste of time and capacity. As phrased by a senior policy advisor of the Federal Police (3): ‘*If you’re a first line agent, you only have two hands’* and not everything can be a priority for local police officers. Recalling a conversation with the Governor a Belgian Province, the investigative journalist quoted the Governor as follow: ‘*There are no structural solutions, and the actions undertook are useless vis à vis chore of the problem. We’re regulating the phenomena, we’re tranquilizing the population, avoiding loitering, long-lasting bivouacs and phenomenon of violence, we’re showing the population that the police is there and watches*’. Regarding the interventions of first line officers, such arrests can lead to logistic (when it comes to transportation), sanitary and security issues when it comes to placing the migrants in small police zone offices for hours. A local police investigator (2) questioned whether there was still a place for empathy towards migrants in transit in the circumstances described above and expressed his dissatisfaction as follow: ‘For us it is rare but indeed, for a small police force, it’s f******g hell to be suddenly confronted with these people’.

The ‘hell’ situation on the one hand refers to police officers being ‘outnumbered’ by migrants, which was not seen as optimal, and on the other hand to the conditions in which migrants themselves had to be placed in small local police offices, which are not appropriately designed to accommodate migrants. Nonetheless, on the question of whether empathy could still be found with regard to logistic issues faced by police officers and in line with the findings of Vega (2018) on legitimation narratives of US Border Agents, we can observe, from an emotional perspective, a form of detached professionalism and strict boundary making between the respondent and the migrants in this answer (Aas & Gundhus, 2015). The same comment of boundary making and distance can be made about the police inaction highlighted above. Moreover, the feeling of danger of being ‘outnumbered’ by migrants can also prevent feelings of compassion and result in the strict application of bureaucratic procedure (Vega, 2018: 2554). It is also important to question the extent to which bringing forward logistic/capacity issues and using narratives of ‘care control’, as the detention conditions are depicted as problematic from a care lens by the respondent, can allow bureaucrats to continue to perform their tasks in an uncritical manner (see Vega, 2018; Aliverti, 2020).

### Passing the buck and shifting the blame

Both Prosecutor 1 and Prosecutor 4 emphasize the complementarity and the expertise of the local and federal police forces, which are supposed to function in an integrated manner at two levels in dealing with the migrant smuggling phenomenon. Nonetheless, despite examples where the collaboration can be efficient and structural, it is not necessarily the case everywhere. As Respondent 2 of the Foreigner’s Office underlines: ‘*The police forces are working against each other or have different interests, especially in the Maximilian Park. Everyone tries to give the hot potatoes to someone else.*’ Issues of capacity and priority were mentioned to justify the fact that police officers could turn a blind eye and look to other directions when they saw a suspicious smuggling situation. The Foreigner’s Office’s respondents (2 and 3) and the Federal Police Investigator explained that the local police were pointing fingers at the federal police and vice-versa when it comes to tackling the issue. Going further, Prosecutor 4 and the Federal Police Investigator both mentioned the mentality to pass the buck in the migrant smuggling field because ‘*the powers are scattered (…) and people look at each other all the time*’ and refuse to ‘*work on it anymore because the others don’t work on it either*’ (Prosecutor 4). The respondent explained that if people didn’t know who to look at anymore, ‘*then they put it on the English or on the country of origin’*. The Federal Police Investigator felt with a certain fear that he had to answer the following question more often: ‘*why put so much money in a group of 15 investigators in Brussels for an issue that is not our problem*’. Yet, both respondents considered firmly that once you have individuals dying and being abused in the Belgian territory, then it becomes ‘*our problem’*. Mentioning the case of the EU and the need for a structural approach, the Respondents of the Foreigner Office (1 and 2) explained that Belgium was at the end of the ‘*transit chain*’, but that overall: ‘*Member states are happy when people leave their territory illegally. How, when and what? As soon as possible and it’s no longer our responsibility, period.*’ The Federal Police Investigator pinpointed similar reasons for individuals to end up in the Maximilian Park as they were pushed away from both the British and the French. Respondent 3 from the Foreigner’s Office has a rather grim outlook on the future:

‘*If it's even a challenge to agree at the Belgian level. And that's Europe's big challenge, to provide an answer within ten years. What are we going to do then? We have to be ready. We have to agree because if we don't agree... I think it's a disaster right now. I don't see it getting any better. I don't see any dialogue either. There's dialogue between two member states but there's no dialogue in Europe. It's far too complex and too difficult*.’

The image that the respondents paint is, unfortunately, not unique to the Belgian case. The challenges of working in multi-jurisdictional spaces with unclear lines of responsibility, particularly in the complex relationship between EU bodies, national and local authorities, are known to lead to frequent blame shifting between these different actors (e.g., Van der Woude, 2020; Dörrenbächer & Mastenbroek, 2019). In line with Aas and Gundhus’s (2015) findings on the experiences of Frontex officials, our findings reveal both a considerable level of frustration with the third-way approach, as well as the difficulty of performing policing tasks in multi-jurisdictional areas, where the lines of responsibility become blurred on the ground.

## Conclusion

In the realm of migrant smuggling and human trafficking and considering the blurred boundaries between these phenomena, the tensions highlighted above have concrete consequences on the legal protection (or the lack thereof) given to smuggled migrants. In their work on the relation between anti-trafficking policies (protective oriented) and migration control policies (punitive oriented), van der Leun and Van Schijndel (2016) observed that treatment given to irregular migrants depended largely on the way they entered the system and that smuggled migrants often ended up unprotected even though traces of exploitation were visible in their criminal investigation files. The authors argued that while migration control and anti-trafficking policies are interrelated, they nevertheless exist alongside each other following their own courses, which have detrimental impact on potential victim protection if these sets of policies and the relationship between them are not structurally and fundamentally re-examined. This article responds to that call for re-examination by looking closely at the third-way approach in Belgium, an approach aimed to provide victims of aggravated forms of migrant smuggling similar legal protection as victims of human trafficking. By listening to and critically engaging with (frontline) implementers and examining their experiences and perspectives (following Loftus 2015), this article has provided a first understanding of why the protective third-way approach does not seem to be used as much in practice. Despite the rich insights provided through our interviews, an important limitation – and thus also recommendation for further research – is that we did not speak with migrants directly to ask about their motivations and reasoning to not make use of the protective status offered through the third-way approach. This would be important to do, as the reflections of our respondents seem to illustrate the agency that migrants can have and can take in deciding how to shape their (legal) journey, which might result in well-intended policies and procedures completely missing their aim.

Our data furthermore revealed a rather ‘Janus-faced’ image of how the respondents view the state of vulnerability of the potentially smuggled migrants: Whereas on the one hand this is acknowledged through their general reflections on the interwovenness of human trafficking and migrant smuggling, when urged to reflect a bit more on the practical applications of the third-way approach, respondents seem to have a hard time stepping out of straight-jacket of the legal dichotomy between the two phenomena. While our respondents predominantly seemed to ‘blame’ migrants for not wanting to make use of the third-way approach, this Janus-faced response indicates that those who are implementing the law and the organizational context in which they operate are also playing a crucial role in whether or not migrants get to access the third-way approach protective status. As mentioned by Vega (2018) and Fassin (2005, 2011), the role of the moral economy of government bureaucracies in which state agents operate cannot be underestimated, as it can contribute to a tension between the normative values and ideals that are dominant within the different realities in which these state agents operate and the emphasis that is placed within these different realities on either compassion or repression.

This tension is not just visible and tangible in Belgium, but throughout the EU and particularly around asylum (also see Bloch & Schuster, 2002). On the one hand European countries and societies are tooting the horn of tolerance, human rights, and solidarity, but on the other hand they are actively creating and maintaining a migration control apparatus that seems to foster ‘bureaucratic indifference to human needs and suffering’ (Herzfeld, 1992: 1). What also doesn’t help in this regard is the complexity of having to operate and implement norms in a fragmented and multi-layered national and European institutional context. The interviews uncovered issues and challenges linked to institutional capacity, sensibilization of frontline implementers and a tendency to evade one’s responsibility by passing it on to (other) actors on the local, national (federal) or at the European level. While legal frameworks on human trafficking and migrant smuggling are harmonized at the European level, the difficulties surrounding their implementation and the lack of uniformed approach, especially on migration matters from one member states to another has to be underlined (see Barbero, 2020). This finding is in line with Dörrenbächer and Mastenbroek’s (2019) observations regarding the implementation of the 2003 Asylum Reception Condition Directive in France, Germany in the Netherlands as well as Van der Woude’s (2020) study of the implementation and enforcement of article 23 of the Schengen Borders Code. Both studies illustrate that the attribution of discretion to practical implementers forces implementers on the ground to determine the eventual outcomes of EU law, which can, and will, result in great differences between member states.

Policing and managing the borderlands in a way that reconciles worries about national security with concerns about the vulnerability of individuals on the move remains a complex challenge, not just to the European Union, but to many countries across the globe. The Commissioner for Human Rights within the Council of Europe signaled the lack of assistance devoted to migrants within Europe as a risk factor which makes them ideal targets for exploitation (Mijatovic, 2019). Considering the particular vulnerability of migrants during their fragmented migration journeys, this last concern highlights the importance of rethinking structurally and holistically the relationship between migration control policies and anti-trafficking policies and the inherent tension existing between them.

## Footnotes

1 See Directive 2011/36/EU; Directive 2004/81/EC; Council of Europe Convention on Action against Trafficking in Human Beings (16 May 2005); Directive 2002/90/EC; Framework Decision 2002/946/JHA.

2 The authors chose the term ‘third-way’ because the Belgian model proposes an alternative between two crimes that are considered completely distinct in international legislations.
